# Translation elongation factor eEF1A2 is a potential oncoprotein that is overexpressed in two-thirds of breast tumours

**DOI:** 10.1186/1471-2407-5-113

**Published:** 2005-09-12

**Authors:** Victoria AL Tomlinson, Helen J Newbery, Naomi R Wray, Juliette Jackson, Alexey Larionov, William R Miller, J Michael Dixon, Catherine M Abbott

**Affiliations:** 1 Medical Genetics, School of Molecular and Clinical Medicine, University of Edinburgh, Molecular Medicine Centre, Western General Hospital, Edinburgh EH4 2XU, UK; 2 Breast Unit Research Group, Western General Hospital, Edinburgh EH4 2XU, UK

## Abstract

**Background:**

The tissue-specific translation elongation factor eEF1A2 was recently shown to be a potential oncogene that is overexpressed in ovarian cancer. Although there is no direct evidence for an involvement of eEF1A2 in breast cancer, the genomic region to which EEF1A2 maps, 20q13, is frequently amplified in breast tumours. We therefore sought to establish whether eEF1A2 expression might be upregulated in breast cancer.

**Methods:**

eEF1A2 is highly similar (98%) to the near-ubiquitously expressed eEF1A1 (formerly known as EF1-α) making analysis with commercial antibodies difficult. We have developed specific anti-eEF1A2 antibodies and used them in immunohistochemical analyses of tumour samples. We report the novel finding that although eEF1A2 is barely detectable in normal breast it is moderately to strongly expressed in two-thirds of breast tumours. This overexpression is strongly associated with estrogen receptor positivity.

**Conclusion:**

eEF1A2 should be considered as a putative oncogene in breast cancer that may be a useful diagnostic marker and therapeutic target for a high proportion of breast tumours. The oncogenicity of eEF1A2 may be related to its role in protein synthesis or to its potential non-canonical functions in cytoskeletal remodelling or apoptosis.

## Background

Breast cancer is the most common cancer in females worldwide; there are an estimated 1 million new cases per year [1]. The identification of changes in gene expression in breast tumours relative to normal surrounding tissue is clearly of great importance in terms of prognostic indicators and therapeutic targets.

The translation elongation factor eEF1A2 was first identified as a tissue-specific variant of eEF1A1 (formerly known as EF-1α) in the early 1990s [2,3]. The two forms of eEF1A are encoded by separate loci, but the resulting proteins are 92% identical and 98% similar. Whereas eEF1A1 is widely expressed, eEF1A2 is normally expressed only in neurons and muscle [3-5]. The first specific evidence implicating eEF1A2 in tumourigenesis came in 2002 when Anand et al [6] showed that eEF1A2 was expressed in 30% of ovarian tumours, but not in normal ovary. The genomic region to which eEF1A2 maps, 20q13, had been known for many years to be amplified in a high proportion of ovarian and breast tumours [7]; [8], but the *EEF1A2 *gene maps closer to the telomere than the region previously implicated. Anand et al showed that 14/53 tumours had amplifications of the region surrounding *EEF1A2 *[6]. In the same paper, forced expression of eEF1A2 in cells was demonstrated to confer tumourigenic properties on NIH3T3 cells, and to give rise to tumours in xenografted nude mice. Although 20q13 amplification is commonly observed in breast cancer, there has as yet been no evidence for overexpression of eEF1A2 in breast tumours. However, eEF1A1, the widely-expressed isoform, was recently shown to be upregulated in the infiltrating edge of invasive breast tumours compared with the tumour core by microarray analysis of laser microdissected material, confirmed by immunohistochemistry [9]. In this analysis the antibody used was one that detects both eEF1A1 and eEF1A2 with equal intensity, so it is conceivable that eEF1A2 contributes to this pattern of expression.

We have generated antibodies (Newbery et al, in preparation) that allow us to distinguish between the highly related isoforms eEF1A1 (which is expressed in normal breast) and eEF1A2 (which is thought to be expressed only in muscle and neurons). Using these isoform-specific antibodies, we show that eEF1A2 expression is barely detectable in normal human breast tissue, but that the gene is moderately to strongly expressed in 63 % of breast tumours examined. Furthermore, there is a strong correlation between eEF1A2 overexpression and estrogen receptor (ER) positivity.

## Methods

### Quantitative Real-time Reverse Transcription-PCR (RT-PCR)

Breast cancer samples were obtained in the Edinburgh Breast Unit (Western General Hospital, Edinburgh) with patients' informed consent and ethical committee approval. Biopsies were snap frozen and stored in liquid nitrogen until RNA extraction. Before RNA extraction the frozen tissue was defrosted and stabilized in RNA-later-ICE reagent (Ambion). Total RNA was extracted with RNeasy-mini columns (Qiagen).

Amount and purity of RNA were evaluated by spectrophotometry. RNA integrity was confirmed by agarose gel electrophoresis. Total RNA was isolated from tumour and normal tissue using Qiagen RNeasy kits (Qiagen). RNA was treated with DNase using DNAfree kit (Ambion, Cambridgeshire) and 1 μg was used for RT-PCR using Retroscript kit (Ambion, Cambridgeshire, UK). TaqMan Assay-on-Demand gene expression pre-designed primer and probe sets from Applied Biosystems, Cheshire, UK were used for EEF1A2 (Assay # Hs 00157325 ml) and glyceraldehyde-3-phosphate dehydrogenase (GAPDH; control; Hs 99999905 ml). In a 10 μl reaction volume per well of a 394-well plate, 0.5 μl of primers, 5 μl of TaqMan PCR Master Mix, no AmpErase UNG 10×, and 4.5 μl of diluted cDNA were added (Applied Biosystems, Cheshire, UK). Real-time RT-PCR and the quantification of RT-PCR products were performed and the products analyzed using an ABI Prism 7900HT Sequence Detection System, and the appropriate software (SDS3.1) according to the manufacturer's instructions (Applied Biosystems, Cheshire, UK).

### Western blots

Protein lysates from cell lines were prepared using previously published protocols [10]. Western blot analyses were carried out using standard protocols. The blots were incubated with primary anti-eEF1A2 rabbit antibody and primary anti-eEF1A1 sheep antibody diluted 1:200 in blocking solution, as well as primary anti-glyceraldehyde-3-phosphate dehydrogenase polyclonal mouse antibody (Chemicon International, Hampshire, UK) diluted 1:10000. Blots were then incubated in the appropriate horse radish peroxidase conjugated secondary antibody (Dako Cytomation, Cambridgeshire, UK) at 1:500. Detection was performed using enhanced chemiluminescence detection kit (Amersham Biosciences, Buckinghamshire, UK).

### Immunohistochemistry

Specimens of normal and cancerous tumours were obtained with informed consent and local ethical committee approval from patients undergoing surgical treatment at the Royal Infirmary of Edinburgh and Western General Hospital, Edinburgh. A breast tumour histoarray (CB2) produced by SuperBioChips (AMS Biotechnology, Oxfordshire, UK) was also used. Formalin fixed, paraffin embedded sections of human normal tissue and tumour tissue were deparaffinized with xylene, rehydrated, treated with picric acid and microwaved in citric acid pH6. Slides were blocked in a 1:5 dilution of sheep serum for 30 minutes at room temperature. Primary anti-eEF1A2 rabbit antibodies were used at a concentration of 1:10 diluted in PBS, for 40 minutes at room temperature and secondary goat anti-rabbit IgG biotin conjugated antibody (Dako Cytomation, Cambridgeshire, UK) was used at 1:200 at room temperature for 30 minutes. Slides were incubated with StreptABC complex/HRP (Dako Cytomation, Cambridgeshire, UK) at room temperature for 30 minutes and in diaminobenzidene (Sigma Fast DAB, Sigma, Dorset, UK) for 2 minutes at room temperature. Finally slides were counterstained in haematoxylin, dehydrated and mounted in pertex.

### Immunohistological scoring methods

The breast tumour sections and normal breast sections (CB2, SuperBioChips, AMS Biotechnology, Oxfordshire, UK) were scored as weak, moderate and strong staining for eEF1A2. Weak staining was considered as background since this level of staining is seen in normal tissue. Stromal tissue was negative in all cases. Blind scoring was carried out by two independent researchers. Two slides were analysed, representing different levels within tumours, and each of these was stained with a different antibody to eEF1A2. Almost perfect correlation was seen between the two slides.

### Statistical methods

Fisher's exact test was used to test for associations between negative and weak eEF1A2 expressing tumours or moderately and strongly overexpressing tumours with ER positivity. For breast tumour Quantitative Real-time RT-PCR data, a two-sample t-test allowing for difference in variance between the two samples was used to test the difference between the mean standardised quantity of RNA for the ER-positive and ER-negative groups. P values that were less than or equal to 0.05 were considered significant.

## Results

### Expression analysis in breast tumours

**Figure 1:**
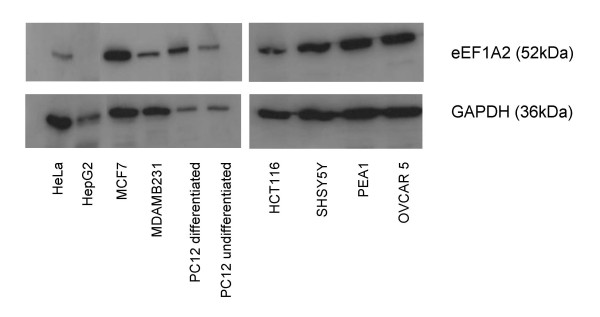
Western blot analysis using an anti-eEF1A2 antibody on a range of cell lines. The loading control is GAPDH.

**Figure 2:**
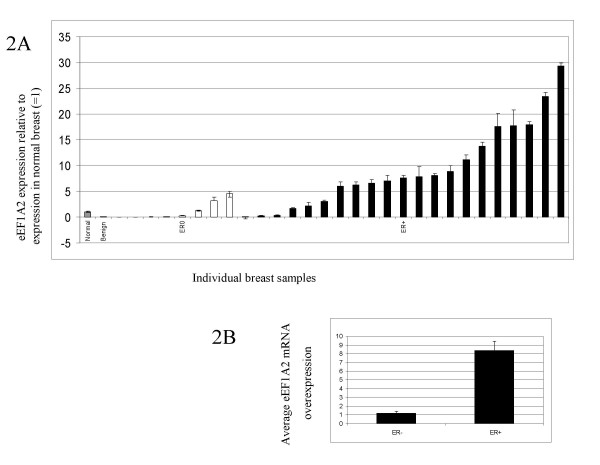
**(A)** Real-time RT-PCR analysis of RNA from breast tumours. Each block on the × axis represents a different tumour. The amount of eEF1A2 message is shown normalised to GAPDH and expressed relative to the level of expression in the normal breast RNA samples (=1). ER-negative tumours are shown in white, ER-positive tumours are shown in black. The difference in mean expression between ER-positive and ER-negative samples is 7.2 units (p = 0.0087), 95% Confidence Interval 2.0 to 12.4 units. **(B)** Average standardised RNA levels in ER-negative and ER-positive breast tumours. This difference is significant (P = 0.0087, t-test).

Since 20q13.3 amplification is commonly seen in breast tumours as well as ovarian tumours, and because analysis of the SAGE database at NCBI indicated that eEF1A2 was more highly represented in breast tumours than in normal breast tissue (unpublished observations) we decided to examine eEF1A2 expression in breast tumours at both the RNA and protein level. Initially, we used Western blotting with an anti-eEF1A2 antibody to examine expression in a number of commonly-used cell lines. The anti-eEF1A2 antibody was raised against a peptide that differs significantly between eEF1A1 and eEF1A2 (Newbery et al, in preparation); specificity was confirmed by lack of signal from tissues taken from wasted mice which have a null mutation of eEF1A2 [5]. The majority of transformed cell lines showed high levels of expression of eEF1A2 (Figure 1); in addition, it has previously been shown that NIH3T3 cells do not express eEF1A2 except when they become confluent [2,6]. We therefore chose not to place any emphasis on the analysis of breast cancer cell lines as opposed to primary tumour samples since eEF1A2 expression seems to be a common property of transformed cells, rather than being specific for a tumour type. Instead, we carried out real-time quantitative RT-PCR of RNA samples from breast tumours. The results obtained are shown in Figure 2A. It can be seen that whereas extremely low levels of expression are detected in RNA samples from normal breast and from a benign breast tumour, most malignant tumour samples showed moderate to high (up to 30-fold higher than normal breast) expression levels. On average, the estrogen receptor (ER)-negative tumours showed only 1.2 times higher expression than the normal sample whereas ER-positive tumours had 8.4 times higher expression (Figure 2B). The difference in eEF1A2 expression levels between the estrogen receptor negative tumours and estrogen receptor positive tumours is 7.2 units (P = 0.0087, t-test, 95% confidence interval 2.0 to 12.4 units).

**Figure 3:**
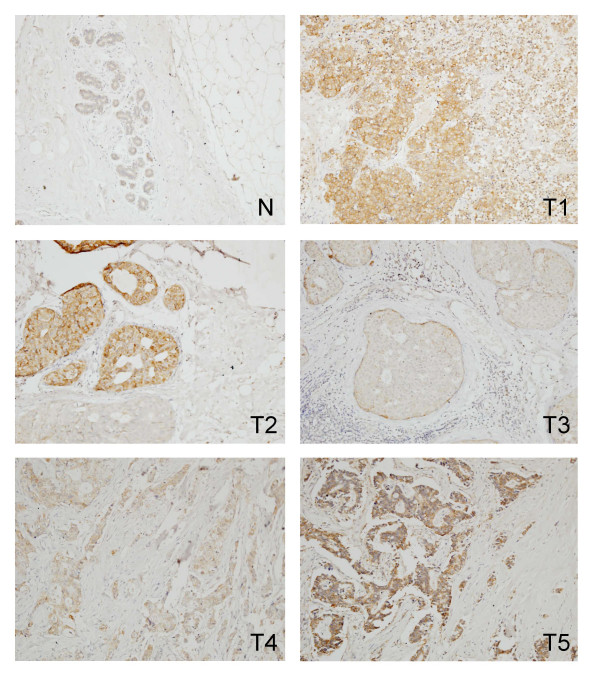
Immunohistochemistry of eEF1A2 in the breast. The panel labelled N shows the antibody staining weakly in a normal breast section. Panels T1 to T5 show breast tumours staining strongly with the anti-eEF1A2 antibody. Magnification ×10.

No protein extracts were available from these tumours for Western blot analysis so we then examined expression of eEF1A2 by immunohistochemistry on a commercial tissue array of normal and tumour breast samples using the anti-peptide antibodies described above. The array contained sections from 46 cases of cancer and 7 normal breasts; the results obtained are shown in Figure 3. None of the normal breast sections showed any more than faint staining. No stromal staining was observed and tumour staining within a sample was near-uniform. Of the tumour samples, 5 showed strong expression of eEF1A2 (11%) and 22 showed moderate expression (48%). The remainder appeared to have no more staining than normal breast. None of the three lobular carcinomas on the slide showed any eEF1A2 overexpression. There was no significant correlation between eEF1A2 expression level and tumour grade or lymph node positivity (data not shown). The tumours had all been previously assessed for p53 status; there appeared to be an association between overexpression of eEF1A2 and wild-type p53, but this was not statistically significant. The tendency to association may be a reflection of the significant association between ER positivity and p53 negativity (p = 0.012) in these tumour samples. Only four out of the 22 ER-negative tumours showed staining beyond background levels and none had strong staining. We found a significant association between eEF1A2 overexpression (scored as moderate/strong) and ER positivity (P = 0.016, Fisher's exact test). We then went on to examine 16 tissue sections from ER positive breast tumours obtained from patients at the Western General Hospital. Of these, 13 showed moderate or strong staining with the anti-eEF1A2 antibody. Overall then, 40 out of 63 breast tumours (63%) examined by immunohistochemistry showed significant overexpression of eEF1A2.

## Discussion

We have shown that the putative oncogene eEF1A2 is upregulated in a high proportion of breast tumours. This upregulation is considerably more significant in ER-positive tumours. There is little or no detectable expression of eEF1A2 in normal breast tissue. It is not yet known whether this overexpression results from amplification of the *EEF1A2 *gene in all cases; in the study of ovarian tumours by Anand et al [6] at least one tumour showed overexpression in the absence of gene amplification, suggesting that there are other mechanisms by which the gene can be upregulated. There is a strong association between ER-positivity and eEF1A2 overexpression which is worthy of further study.

There also appears to be a weak correlation between the absence of p53 mutations and eEF1A2 overexpression. It is possible that eEF1A2 is not upregulated in tumours with p53 mutations because wild-type p53 is required for expression of eEF1A2 in certain cell types; it has been shown that p53 can upregulate expression of eEF1A1 [11], and the p53 binding sites identified in the gene encoding eEF1A1 are shared with that encoding eEF1A2 (unpublished observations). On the other hand it is conceivable that upregulation of eEF1A2 expression rather than p53 mutation is an alternative route for tumours to evade apoptosis in certain cancers.

The basis for the oncogenicity of eEF1A2 is still unclear. We, like Anand et al, have shown that the levels of eEF1A1 in tumours which over-express eEF1A2 are unchanged (data not shown), suggesting that these tumours might have a greater capacity for protein synthesis. However, it has been known for many years that eEF1A is in excess over the other components of the translation elongation apparatus [12], so eEF1A is unlikely to be rate-limiting in protein synthesis. eEF1A1 has been shown to determine the susceptibility of a number of independent cell lines to chemical- and UV-induced transformation [13] and has been identified as an actin binding protein in rat breast tumour cells, where it was found to be more highly expressed in metastatic than non-metastatic cells [14]. It is not yet clear whether these properties are shared with eEF1A2, but the availability of specific antibodies that distinguish between the two isoforms should allow us to shed light on this. One hypothesis is that the non-canonical ("moonlighting") properties of eEF1A1 [15] and eEF1A2 differ so that, for example, the way eEF1A2 interacts with the cytoskeleton might differ from that of eEF1A1 and affect the properties of cells which are expressing high levels of both isoforms. It has been shown, for example, that forced overexpression of eEF1A affects the cytoskeleton in both *S. pombe *and *S. cerevisiae *[16,17]. Alternatively, it has been shown that eEF1A1 and eEF1A2 differ in terms of their response to apoptotic agents [18]; the finding that eEF1A2 is anti-apoptotic, at least in certain conditions, has obvious implications for the possible role of eEF1A2 in tumourigenesis. The observation that eEF1A2 expression is seen in the majority of cell lines, regardless of the tissue of origin, suggests that eEF1A2 expression may be triggered by the general process of transformation. This idea is strengthened by the fact that most of the few cell lines which do not express eEF1A2 tend to be untransformed, such as NIH3T3 cells (Figure 1).

The presence of increased levels of eEF1A2 in breast tumours may provide a useful new diagnostic marker. Further, eEF1A2 may prove to be a feasible target for therapeutic intervention. It has already been shown that growth-factor mediated eEF1A1 expression can be blocked with anti-EGF antibodies [19]; it would be of interest to examine the response of eEF1A2 to similar antibodies. Investigations into non-canonical functions of eEF1A molecules may shed new light on mechanisms of oncogenicity.

## Competing interests

The author(s) declare that they have no competing interests.

## Authors' contributions

VT carried out the molecular analysis, Western blots and immunohistochemistry. HN isolated and characterised the antibodies. NW carried out the statistical analysis. AL made the RNA. JJ provided some of the tissue sections. WM and JMD collected the patient material. CA conceived the study and wrote the manuscript.

## Pre-publication history

The pre-publication history for this paper can be accessed here:
